# Cutaneous leishmaniasis in Qasr-e Shirin, a border area in the west of Iran

**DOI:** 10.14202/vetworld.2018.1692-1697

**Published:** 2018-12-17

**Authors:** Yazdan Hamzavi, Naser Nazari, Nahid Khademi, Keivan Hassani, Arezoo Bozorgomid

**Affiliations:** 1 Department of Medical Parasitology and Mycology, Faculty of Medicine, Kermanshah University of Medical Sciences, Kermanshah, Iran; 2 Department of Disease, Kermanshah Health Center, Kermanshah University of Medical Sciences, Kermanshah, Iran; 3 Students Research Committee, Kermanshah University of Medical Sciences, Kermanshah, Iran

**Keywords:** cutaneous leishmaniasis, Iran, Kermanshah, prevalence

## Abstract

**Aim:**

The prevalence of cutaneous leishmaniasis (CL) is growing in Iran, and new sources of the disease have been found in the country. The purpose of this study was to describe the epidemiology of CL in Qasr-e Shirin County, Kermanshah Province, West of Iran. Qasr-e Shirin is located near the Iran-Iraq border, and several million pilgrims pass through this area to Iraq every year.

**Materials and Methods:**

A descriptive cross-sectional study was carried out for active case detection from April 1, 2014, to March 31, 2015. All individuals with suspicious lesions were identified through house-to-house visits in the aforementioned area. A questionnaire was filled out for everyone with clinical suspicion of CL. A diagnosis was made based on parasitological examination of the lesion exudate. The annual incidence and prevalence of CL were estimated.

**Results:**

In total, 5277 individuals were randomly screened for the presence of active lesions or scars suspected of CL. The overall prevalence of CL in the County was 4.8% (60 cases had active lesions and 194 cases had scars). All age groups were affected, but the highest incidence of CL was seen in the age group 20-29 years (1.9%). The incidence and prevalence were higher in women than men (5.5% vs. 4%). Most cases (45.6%) had a single lesion or scar and 44 (17.4%) patients had five or more lesions or scars.

**Conclusion:**

The incidence of CL in Qasr-e Shirin County is high. There is a need for further studies on reservoirs and vectors of leishmaniasis in this area. The results of such studies help to perform more efficient and timely spraying against phlebotomine sandfly or detect and decrease the animal reservoir population.

## Introduction

Cutaneous leishmaniasis (CL) is a health issue at the global scale, and the problem is even more serious in tropical and subtropical regions. The disease has affected about 12 million in the world, and its expansion has recently passed the threshold of 88 countries. About 350 million people are at the risk of this disease, and 1 million new cases are detected every year [1]. The disease appears as lesions on the skin, and a scar is left when the lesions are healed. It is caused by a single-celled parasite called *Leishmania*, which require a female phlebotomine sandfly for transmission [2]. Determining the endemicity and epidemiology of the disease in every region is one of the main elements in programming for disease control projects. The endemicity of leishmaniasis is classified into three groups of hyperendemic (85% of new cases are in children aged 0-6 years, and 25% of the affected population is below 1 year old), hypoendemic (20% of new cases are <15 years old), and mesoendemic (the rate of new cases varies between the two previous extremes) [3]. Every year, about 30.9 new cases of CL in 100,000 population are detected in Iran; therefore, Iran is known as one of the main sources of CL in the world [4]. The prevalence of CL is growing in Iran, and new sources of the disease have been found in the country. The first introduction of CL occurred during 1983-1997 in Southwest of Iran and then expanded toward the center and the eastern between 1998 and 2013 [4]. Leishmaniasis is a significant public health concern in Iran’s neighboring countries in the east and west, where civil wars have affected the economy and everyday life in the past few decades [5,6].

Kermanshah Province is located in the middle of the western part of Iran and has a border of about 250 km with Iraq to the west. The Khosrawi border is one of the official and international terminals of Iran, located in Qasr-e Shirin County and each year several million Iranians pass through this area to Iraq (a focus of rural leishmaniasis) for religious and employment purposes. Travelers are not only at significant risk of acquiring leishmaniasis, but they also contribute to its spread to other regions. In an analytical-descriptive study, about 47% of the total cases of CL in Kermanshah Province were seen in Qasr-e Shirin district (2011-2012) [7].

Therefore, due to the prevalence of CL and the strategic location of the region, the present study was conducted to investigate the epidemiological indices of CL and also the form and level of distribution of the disease in Qasr-e Shirin County and its suburbs.

## Materials and Methods

### Ethical approval

This paper was ethically approved and funded (with the code 92355) by Kermanshah University of Medical Sciences (KUMS). Before collecting the samples, informed consent was obtained from all participants.

### Study location and participants

**Figure-1:**
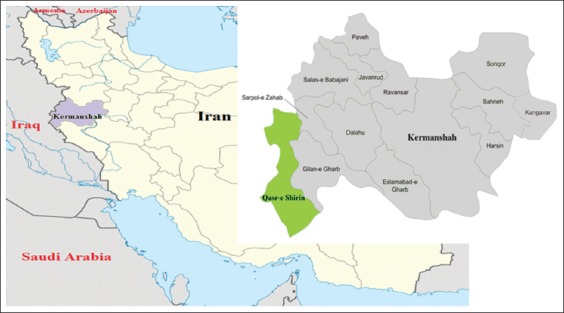
Map of Iran, showing the geographical location of Kermanshah Province and the County of Qasr-e Shirin, our study area (green).

This descriptive cross-sectional study was carried out through active detection of CL cases in Qasr-e Shirin County, Kermanshah Province, Iran, from the beginning of April 2014 to the end of March 2015. Kermanshah Province borders Ilam Province in the south, Hamadan Province in the east, and Iraq in the west with about 250 km of international border. Qasr-e Shirin County is located in the west of Kermanshah Province (Figure-1). It has a temperate climate in the winter but a hot and dry climate in the summer.

Based on the previous study in Iran mentioning the prevalence of 0.26% for CL [7], with confidence level of 90% and a margin of error of 1%, the minimum sample size required was 5141 subjects, which was increased to 5277 to allow for dropouts. To select the cases, random cluster sampling was used in all geographical regions of the city and nearby villages. In each cluster form the villages of Syed Ayaz, Jegarloo Agha Brar, Syed Ahmad, Syed Saeed, Ney Pahn-e Abdollah, Garaweh Dow, Bareh Boo Anjiriyan, and Ney Pahn-e Seyfollah and five regions of the city sampling were conducted according to the proposal.

### Study design

The researchers visited all houses in the County and villages in the study area to find any suspicious cases. Sampling was done using a lancet or a vaccinostyle. The lesion exudates of the suspected patients were smeared on slides, fixed in methanol, and stained using the Giemsa stain. All stained smears were examined microscopically for *Leishmania* amastigotes. In addition, a questionnaire was filled out for any person suspicious of CL including various factors such as age, sex, occupation, site of lesion, or scar. All the family members in the study areas were examined. The health staff also visited the population in the following months only to record new cases. Since determining the incidence (seasonal or annual) was one of the objectives of the study, the questionnaire was filled out in the middle month of each season based on the Iranian calendar (May, August, November, and February).

### Statistical analysis

Statistical analysis was performed by the software SPSS version 16.0 (SPSS Inc., Chicago, IL, USA). Pearson’s Chi-square test (*χ*^2^) was used to determine any significant difference between demographic factors and CL. p<0.05 was considered significant.

## Results

**Figure-2:**
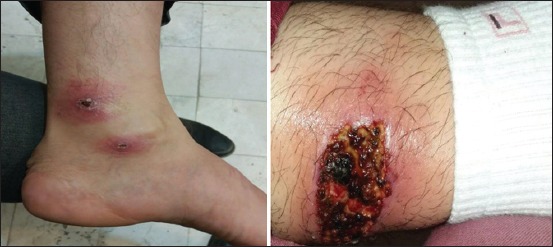
Morphology of skin lesions in patients with cutaneous leishmaniasis from Qasr-e Shirin, Iran (Photo by Yazdan Hamzavi) .

In total, 5277 individuals were randomly screened for the presence of active lesions or scars suspected CL. During the study period, 254 CL cases (40.6% male and 59.4% female) were identified. Of 254 cases, 60 (23.6%) showed active lesions and 194 (76.4%) had scars (Figure-2). The highest incidence was seen in the age range 20-29 years (1%), and the lowest was observed in individuals <80 years (0.9%). Military staff and children had the highest rate of incidence (5.1% and 2.1%, respectively). The demographic characteristics of the CL patients are summarized in Table-1.

**Table-1:** Prevalence of active lesion and scar in Qasr-e Shirin County (April 2014-March 2015).

Categories	Screened people	CL groups: Number of patients (%)			p-value

		Active lesion	Scar	Total	
Sex					
Male	2543	21 (0.8)	82 (3.2)	103 (4)	0.027
Female	2734	39 (1.4)	112 (4.1)	151 (5.5)	
Age group (years)					
0-9	766	7 (0.9)	6 (0.8)	13 (1.7)	<0.001
10-19	740	8 (1.1)	18 (2.4)	26 (3.5)	
20-29	1095	21 (1.9)	30 (2.7)	51 (4.6)	
30-39	846	9 (1.1)	23 (2.7)	32 (3.8)	
40-49	597	6 (1)	23 (3.8)	29 (4.8)	
50-59	449	5 (1.1)	31 (6.9)	36 (8)	
60-69	334	2 (0.6)	42 (12.6)	44 (13.2)	
70-79	185	1 (0.5)	15 (8.1)	16 (8.6)	
>80	215	1 (0.5)	6 (2.8)	7 (3.3)	
Zone					
Urban	2003	36 (1.8)	23 (1.1)	59 (2.9)	<0.001
Rural	2342	17 (0.7)	159 (6.8)	176 (7.5)	
Nomads	932	7 (0.7)	12 (1.3)	19 (2)	
Occupation					
Housewife	1866	18 (1)	65 (3.5)	83 (4.5)	<0.001
Children	325	7 (2.1)	10 (3.1)	17 (5.2)	
Military	273	14 (5.1)	22 (8.1)	36 (13.2)	
Student	535	5 (0.9)	18 (3.4)	23 (4.3)	
Farmer	703	4 (0.6)	33 (4.7)	37 (5.3)	
Rancher	683	3 (0.4)	10 (1.5)	13 (1.9)	
Other	685	9 (1.3)	36 (5.3)	45 (6.6)	

CL: Cutaneous leishmaniasis

**Figure-3:**
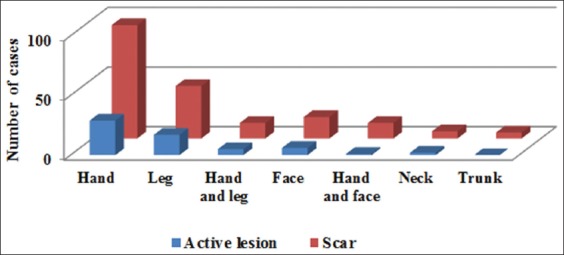
Distribution of cutaneous leishmaniasis in Qasr-e Shirin County by location of lesion.

**Figure-4:**
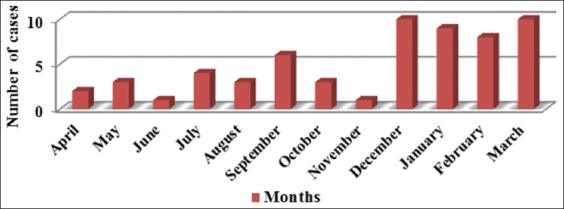
Incidence of cutaneous leishmaniasis cases (active lesions) according to months from 2014 to 2015.

The hand was the most commonly affected site (48.8%), followed by the leg (24%), the hand and leg (7%), and other parts of the body (20.2%) (Figure-3). Most cases (45.6%) had a single lesion or scar, and 44 (17.4%) patients had five or more lesions or scars. The findings showed that the prevalence of the disease was higher in some rural areas of Qasr-e Shirin County such as Ney Pahn, Syed Ahmad, Jegarloo Agha Brar, and Bareh Boo Anjiriyan. The highest incidence was between December and March (61.6%) as shown in Figure-4. Among patients with lesions, only eight cases had a history of travel to other cities in the Province, and 52 cases had no record of traveling. In addition, the results showed that lesions in about 50% of the cases appeared in the past 1-40 days. Moreover, two patients found the lesion on their body 150 days before the examinations.

## Discussion

CL is an important public health problem that extension in regions where environmental factors are suitable for proliferation of both reservoir and the vector. The majority of the CL cases are located in hot dry area [8]. Here, we surveyed the incidence of CL in Qasr-e Shirin County from 2014 to 2015. This is the first epidemiological study of CL using active case detection through house-to-house visits in the west of Iran. Qasr-e Shirin is a moderately warm region with hot and dry summers, an ideal condition for sandflies to breed.

The incidence rate of CL of 1133 per 100,000 people (1.1%) in the present study is higher than a previous provincial study done in 2011-2012 which showed a prevalence of 264.5/100,000 based on passive case reporting [7]. Looking at different parts of the country, a study of the prevalence of CL in 46799 individuals in Abdanan County showed a prevalence of 0.34% [9]. The incidence of CL in Kashan city was found to be 47 per 100,000 habitants in 2016 [10]. In villages of Dehbakry County, examination of 3884 individuals showed a rate of 5.3% for active infections and scars of CL [11]. In addition, Al-Bajalan *et al*. [12] observed that the incidence of CL was 27.5 per 100,000 populations, in a study carried out in Garmian area, Iraq, bordering with Iran. By active case detection, more CL cases may be identified since many patients never seek medical care. Qasr-e Shirin borders Iraq, which suffers civil wars, which might explain the high prevalence of the infection in this area of Iran. Qasr-e Shirin was occupied during the 8-year Iraq-Iran war, and almost all residential blocks were severely damaged or even destroyed. It is believed that rural background, old destroyed buildings, hot climate, reduced rainfall, and shared borders with Iraq might explain the high prevalence of CL in this area of Iran.

Most of the patients in this study were from rural areas. This finding could be due to the abundance of sandfly breeding sites, reservoir hosts, and natural habitats in rural areas. Several reports indicated the occurrence of *Tatera indica* Hardwicke (*Rodentia*: *Gerbillinae*) (Indian jird) as the primary reservoir host of *L. major* in west and south-west of Iran [13,14]. In addition, it is evident that having domestic animals and vegetation in or around the house (characteristic of rural settings) increase the risk of CL [15].

In this study, the hands were the most commonly affected site (47.0%). In zoonotic CL, this pattern of lesion distribution is common. Similar findings have been reported in Libya and Iran [16,17]. In the present study, only a few patients had a history of travel to other regions, so autochthonous transmission occurs in these areas. Accordingly, native and traveler populations are at risk for *Leishmania* infection by the bite of infected female phlebotomine sandfly. As shown in the current study, the female to male ratio was 1.46:1, which is a statistically significant difference (p=0.012). This finding is similar to the results of other studies conducted in Iran [18,19]. In contrast, many studies have reported a higher prevalence in males [20-22].

Many researchers have stated that military personnels are more susceptible to infections than others [23,24]. We also noted that a marked percentage (23.3%) of the patients was military personnel working near the border areas and suburbs of Qasr-e Shirin. Military personnel, due to lack of access to appropriate health facilities and the nature of their profession that requires them to spend most of their time outdoors, have an increased risk of exposure to infected insects. In addition, military personnel usually come from non-endemic areas. The movement of non-immune populations to endemic regions leads to the continuation of the disease transmission cycle.

Although molecular identification of *Leishmania* species was not carried out in this study, *L. major* seems to be the probable etiologic agent of the disease in the region. Consistent with these findings, Nemati *et al*. [25], identified *L. major* as the causative agent of CL in the patients of Kermanshah Province. This finding was also reported in a study by Hamzavi *et al*. [26] using the RAPD-PCR method. In addition, Haddad *et al*. [27] reported that *L. major* is more prevalent than *Leishmania tropica* in Ilam Province, which neighbors Qasr-e-Shirin.

Another notable point is the seasonal distribution of the disease incidence. Similar to the findings of previous studies in Iran, the highest incidence was observed in the autumn and winter, which is a clear sign of rural CL [22,28]. The risk of transmission of the rural type of the disease is higher in the spring, summer, and autumn in some areas when the vector flies are abundant. The wound appears after an incubation period of few months in the autumn or winter. The prevalence of the urban type of the disease is constant throughout the year [29].

The most reliable diagnostic methods for CL are culture or molecular techniques. However, it was not possible to implement these techniques due to lack of equipment. To address this limitation, it should be noted that CL in endemic regions is usually estimated using microscopic observations instead of more reliable methods such as culture and molecular techniques.

## Conclusion

The incidence of CL in Qasr-e Shirin County is high. International travelers, military personnel, and other groups of travelers are increasingly involved in outdoor activities, which increase the risk of leishmaniasis. Therefore, people traveling to this area should receive counseling and appropriate and effective use of personal protection measures to avoid a sandfly bite. This indicates the need for further investigations on the various species of *Leishmania* parasites responsible for CL, reservoirs, and sandfly as the vector of leishmaniasis in these regions for more efficient and timely spraying to control phlebotomine sandfly and spotting animal reservoirs.

## Authors’ Contributions

YH designed the study. NK and KH collected the samples. YH, NN and AB contributed to the parasitological test. YH and NK interpreted the results and analyzed the data. AB prepared the manuscript. All authors read and approved the final manuscript.

## References

[1] Alvar J, Vélez I.D, Bern C, Herrero M, Desjeux P, Cano J, Jannin J, den Boer M;WHO, Leishmaniasis Control Team (2012). Leishmaniasis worldwide and global estimates of its incidence. PLoS One.

[2] Torres-Guerrero E, Quintanilla-Cedillo M.R, Ruiz-Esmenjaud J, Arenas R (2017). Leishmaniasis: A review. F1000Res.

[3] Shirzadi MR (2012). Guideline for Cutaneous Leishmaniasis Care in Iran. The Office of the Zoonotic Diseases, Center for Disease Control, Ministry of Health and Medical Education.

[4] Holakouie-Naieni K, Mostafavi E, Boloorani A.D, Mohebali M, Pakzad R (2017). Spatial modeling of cutaneous leishmaniasis in Iran from 1983 to 2013. Acta Trop.

[5] Salam N, Al-Shaqha W.M, Azzi A (2014). Leishmaniasis in the Middle East: Incidence and epidemiology. PLoS Negl. Trop. Dis.

[6] Hussain M, Munir S, Khan T.A, Khan A, Ayaz S, Jamal M.A, Ahmed I, Aziz S, Watany N, Kasbari M (2018). Epidemiology of cutaneous leishmaniasis outbreak, Waziristan, Pakistan. Emerg. Infect. Dis.

[7] Hamzavi Y, Khademi N (2015). Trend of cutaneous leishmaniasis in Kermanshah Province, West of Iran from 1990 to 2012. Iran. J. Parasitol.

[8] Chelbi I, Kaabi B, Bejaoui M, Derbali M, Zhioua E (2009). Spatial correlation between *Phlebotomus papatasi* Scopoli (*Diptera: Psychodidae*) and incidence of zoonotic cutaneous leishmaniasis in Tunisia. J. Med. Entomol.

[9] Askari A, Sharifi I, Aflatoonian M.R, Babaei Z, Almani P.G.N, Mohammadi M.A, Alizadeh H, Hemati S, Bamorovat M (2018). A newly emerged focus of zoonotic cutaneous leishmaniasis in South-Western Iran. Microb. Pathog.

[10] Moein D, Masoud D, Saeed M, Abbas D (2018). Epidemiological aspects of cutaneous leishmaniasis during 2009-2016 in Kashan city, central Iran. Korean J. Parasitol.

[11] Sharifi I, Poursmaelian S, Aflatoonian M.R, Ardakani R.F, Mirzaei M, Fekri A.R, Khamesipour A, Parizi M.H, Harandi M.F (2011). Emergence of a new focus of anthroponotic cutaneous leishmaniasis due to *Leishmania tropica* in rural communities of Bam district after the earthquake, Iran. Trop. Med. Int. Health.

[12] Al-Bajalan M.M.M, Al-Jaf S.M.A, Niranji S.S, Abdulkareem D.R, Al-Kayali K.K, Kato H (2018). An outbreak of *Leishmania major* from an endemic to a non-endemic region posed a public health threat in Iraq from 2014-2017: Epidemiological, molecular and phylogenetic studies. PLoS Negl. Trop. Dis.

[13] Akhoundi M, Mohebali M, Asadi M, Mahmodi M.R, Amraei K, Mirzaei A (2013). Molecular characterization of *Leishmania* spp. In reservoir hosts in endemic foci of zoonotic cutaneous leishmaniasis in Iran. Folia Parasitol. (Praha).

[14] Javadian E, Dehestani M, Nadim A, Rassi Y, Tahvidare-Bidruni G.H, Seyedi-Rashti M.A, Shadmehr A (1998). Confirmation of *Tatera indica* (*Rodentia*
*Gerbillidae*) as the main reservoir host of zoonotic cutaneous leishmaniasis in the West of Iran. Iran. J. Public Health.

[15] Ramezankhani R, Hosseini A, Sajjadi N, Khoshabi M, Ramezankhani A (2017). Environmental risk factors for the incidence of cutaneous leishmaniasis in an endemic area of Iran: A GIS-based approach. Spat. Spatiotemporal Epidemiol.

[16] Abdellatif M.Z, El-Mabrouk K, Ewis A.A (2013). An epidemiological study of cutaneous leishmaniasis in Al-Jabal Al-Gharbi, Libya. Korean J. Parasitol.

[17] Rostami M.N, Saghafipour A, Vesali E (2013). A newly emerged cutaneous leishmaniasis focus in central Iran. Int. J. Infect. Dis.

[18] Khosravi A, Sharifi I, Fekri A, Kermanizadeh A, Bamorovat M, Mostafavi M, Aflatoonian M.R, Keyhani A (2017). Clinical features of anthroponotic cutaneous leishmaniasis in a major focus, Southeastern Iran, 1994-2014. Iran. J. Parasitol.

[19] Aflatoonian M.R, Sharifi I, Aflatoonian B, Shirzadi M.R, Gouya M.M, Kermanizadeh A (2016). A review of impact of bam earthquake on cutaneous leishmaniasis and status: Epidemic of old foci, emergence of new foci and changes in features of the disease. J. Arthropod Borne Dis.

[20] Araujo A.R, Portela N.C, Feitosa A.P.S, Silva O.A, Ximenes R.A.A, Alves L.C, Brayner F.A (2016). Risk factors associated with American cutaneous leishmaniasis in an endemic area of Brazil. Rev. Inst. Med. Trop. Sao Paulo.

[21] Mogalli N.M, El Hossary S.S, Khatri M.L, Mukred A.M, Kassem H.A, El Sawaf B.M, Ramadan N.F (2016). Clinicoepidemiologic pattern of cutaneous leishmaniasis and molecular characterization of its causative agent in Hajjah governorate, Northwest of Yemen. Acta Trop.

[22] Nazari M, Nazari S, Hanafi-Bojd A.A, Najafi A, Nazari S (2017). Situation analysis of cutaneous leishmaniasis in an endemic area, South of Iran. Asian Pac. J. Trop. Med.

[23] Galgamuwa L.S, Sumanasena B, Yatawara L, Wickramasinghe S, Iddawela D (2017). Clinico-epidemiological patterns of cutaneous leishmaniasis patients attending the Anuradhapura teaching hospital, Sri Lanka. Korean J. Parasitol.

[24] Semage S, Pathirana K, Agampodi S.B (2014). Cutaneous leishmaniasis in Mullaitivu, Sri Lanka: A missing endemic district in the leishmaniasis surveillance system (2014). Int. J. Infect. Dis.

[25] Nemati S, Fazaeli A, Hajjaran H, Khamesipour A, Anbaran M.F, Bozorgomid A, Zarei F (2017). Genetic diversity and phylogenetic analysis of the Iranian *Leishmania* parasites based on HSP70 gene PCR-RFLP and sequence analysis. Korean J. Parasitol.

[26] Hamzavi Y, Nomanpour B, Karaji A.G (2010). Identification of species of *Leishmania* isolated from patients with cutaneous leishmaniasis in Kermanshah;Using RAPD-PCR technique. J. Kermanshah Univ. Med. Sci.

[27] Haddad M.H.F, Ghasemi E, Maraghi S, Tavala M (2016). Identification of *Leishmania* species isolated from human cutaneous leishmaniasis in Mehran, Western Iran using nested PCR. Iran. J. Parasitol.

[28] Khosravani M, Nasiri Z, Keshavarz D, Rafat-Panah A (2016). Epidemiological trend of cutaneous leishmaniasis in two endemic focus of disease, South of Iran. J. Parasit. Dis.

[29] Vazirianzadeh B, Hoseini S, Rezaee S.P, Gardani H, Amraee K (2014). Prevalence of cutaneous leishmaniasis in Ramshir, Iran an epidemiological study. Int. Arch. Health Sci.

